# Microdeletion of 4p16.2 in Children: A Case Report and Literature Review

**DOI:** 10.1155/2022/6253690

**Published:** 2022-04-09

**Authors:** Yanjie Qian, Xiaoying Wang, Wei Tang, Chaochun Zou

**Affiliations:** ^1^Department of Endocrinology, Children's Hospital of Zhejiang University School of Medicine, Hangzhou, China; ^2^Department of Pediatrics, The First People's Hospital of Jiande, Hangzhou, China

## Abstract

Copy number variations (CNV) are thought to play an important role in causing human diseases, including congenital anomalies, psychiatric disorders, and intellectual disabilities. We report here a one-year-old boy presented to our clinic as developmental delay. He presented a birth weight of 4.5 kg, motor delay, mental retardation, mild hypertonia, and some dysmorphic features (mild frontal bossing, hypertelorism, epicanthus, concave nasal ridge, slightly sparse hair, short hands, and mild nail dysplasia). The brain MRI indicated brain abnormalities; the Gross Motor Function Measure-66 score was 23.37; the Gesell test result showed the development quotient was 50, suggesting mental retardation. Chromosomal microarray analysis showed an approximately 97 kb microdeletion at 4p16.2 (4p16.2 CNV), including part of EVC and EVC2 genes, which were associated with Ellis-van Creveld syndrome (EvC) and Weyers acrofacial dysostosis (WAD). This report suggests 4p16.2 microdeletion may be associated with multiple developmental abnormalities, including motor delay and mental retardation.

## 1. Introduction

Copy number variations (CNV) are structural variations of the DNA sequence greater than 50 bp in size, including deletions and duplications [1]. Mounting evidence suggests a role of CNV in causing human diseases, including congenital anomalies, psychiatric disorders, and intellectual disabilities. Located in 4p16.2, EVC and EVC2 genes are arranged in a head-to-head configuration and encode EVC and EVC2 proteins [2]. Mutations in EVC or EVC2 gene are associated with two syndromes, Ellis-van Creveld syndrome (EvC, OMIM 225500) and Weyers acrofacial dysostosis (WAD, OMIM 193530). EvC is an autosomal recessive chondro-ectodermal dysplasia characterized by short stature, short limbs, growth retardation, postaxial polydactyly, nail dysplasia, and cardiac malformations (in 50–60% of cases). WAD is an autosomal dominant condition presenting a semblable but milder phenotype compared to EvC. Although many cases involving variants in EVC or EVC2 gene have been reported, the genotype-phenotype correlations in EVC or EVC2 associated disorders are still undiscovered.

Herein, we report on a patient carrying an approximately 97 kb microdeletion at 4p16.2 (4p16.2 CNV) including part of EVC and EVC2 genes. He manifested prominent delayed motor and brain development, with some mild features of EvC and WAD.

## 2. Case Presentation

The one-year-old Chinese boy was referred to our unit with the main complaint of developmental delay. He was G1P1 of nonconsanguineous parents and was born at 39 weeks of gestation with a birth weight of 4.5 kg (>97th percentile). The parents had normal height and weight, and the mother denied a diagnosis of diabetes mellitus or gestational diabetes mellitus. The pregnancy was uneventful. He was born through vaginal delivery with an Apgar score of 6 at 1 min. The amniotic fluid was mildly contaminated. He had neonatal jaundice on day 3 of life and was treated with phototherapy. When he was 6 months old, he reported to the local hospital with the chief complaint of motor delay. It was noticed that the brain MRI showed enlarged bilateral prefrontal space and sulci and white matter volume loss. At 7 months old, he was reported to wobble when grasping the object, with hands being stiff. He had difficulty sitting alone and rolling over. Mildly increased muscle tone, adducted thumbs, and standing on toes were noticed. The brain MRI showed agenesis of corpus callosum and slightly widened subarachnoid space in bilateral frontal lobes. The Gross Motor Function Measure-66 score was 23.37 (confidence interval: 19.45–27.29). The Gesell test result showed the development quotient was 50, indicating a retarded brain development. Electroencephalogram was normal. His muscle tone kept mildly increased. At 1 year old, he was presented to our unit as he was still unable to crawl or keep balance when sitting alone.

**Figure 1:**
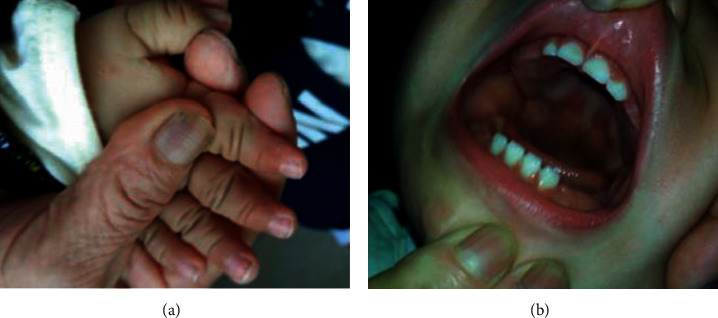
Photos of the current patient. (a) The short hand with mild nail dysplasia. The palm length of his left hand was 4.2 cm and the finger length of his left hand was 2.6 cm, with a total length of 6.8 cm (<10^th^ percentile). The palm length of his right hand was 4.2 cm and the finger length of his right hand was 2.5 cm, with a total length of 6.7 cm (<10^th^ percentile). (b) Oral cavity. No signs of tooth abnormalities or multiple frenula.

On general physical examination, the height was 74 cm (-1SD) and the weight was 9.2 kg (-1SD). Some dysmorphic facial features were observed, including mild frontal bossing, hypertelorism, epicanthus, and concave nasal ridge. The palm length of his left hand was 4.2 cm, and the finger length of his left hand was 2.6 cm, with a total length of 6.8 cm (<10^th^ percentile). The palm length of his right hand was 4.2 cm and the finger length of his right hand was 2.5 cm, with a total length of 6.7 cm (<10^th^ percentile). Mild nail dysplasia and slightly sparse hair also were noticed; however, no signs of congenital heart defects, tooth abnormalities, multiple frenula, or postaxial polydactyly were found (Figures 1(a)–1(b)). Liver and kidney function, thyroid function, CMV DNA and IgM, serum 25-hydroxyvitamin D level, and gas chromatography/mass spectrometry (GC/MS) screening for organic acids were all in normal range or negative.

**Figure 2:**
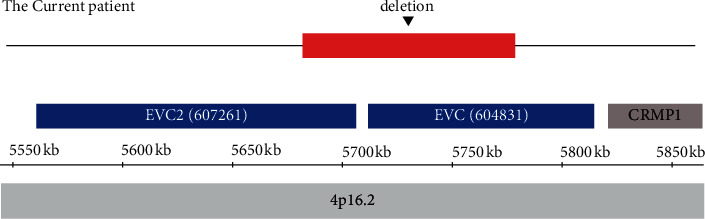
The result of CMA revealed an approximately 97 kb microdeletion at 4p16.2 (5,683,080–5,780,476), which included part of EVC and EVC2 genes.

Chromosomal microarray analysis (CMA, Affymetrix CytoScan® HD Array, GRCh37) was performed and showed an approximately 97 kb microdeletion at 4p16.2 (5,683,080–5,780,476), which included part of EVC and EVC2 genes (Figure 2). Unfortunately, his parents refused further medical assessments including genetic analysis for parents.

## 3. Discussion

A simultaneous deletion or mutation involving both EVC and EVC2 genes is rarely reported, and no reports of a similar or smaller pathogenic deletion were retrieved from the DECIPHER database or the ISCA database.

The current patient had a high birth weight and presented prominent brain and motor developmental retardation. Short hands, slightly sparse hair, mild nail dysplasia, and some dysmorphic facial features also were observed. These manifestations suggested multiple developmental abnormalities, which may be associated with genetic defects. CMA detected an approximately 97 kb microdeletion at 4p16.2 involving part of EVC and EVC2 genes in the current pediatric patient, which made us wonder whether the deleted part of EVC and EVC2 genes could explain the multiple developmental abnormalities. The ectoderm layer and hedgehog signaling play an essential role in central nervous system (CNS) development and are hypothesized to be injured when EVC or EVC2 mutates [3–5]. It is possible that the microdeletion involving part of EVC and EVC2 led to the brain and motor developmental delay in the current patient. Macrosomia and other abnormalities may also be ascribed to the microdeletion because no other obvious cause was found. Although relatively uncommon, several cases reported CNS anomalies in EvC patients [6,7]. Comparing the clinical features of EvC, WAD, and the current patient reported here (Table 1), some manifestations of the patient (short hands, sparse hair, and mild nail dysplasia) were reminiscent of some EvC features or cases, but the lack of specific manifestations of EvC or WAD prevents a clinical diagnosis of EvC or WAD [8–11]. The genotype-phenotype correlations and pathogenesis in EVC or EVC2 mutations associated disorders are still undiscovered; therefore, our case may provide clues for further studies.

In the current case, CMA was performed and detected the 4p16.2 microdeletion in the patient. CMA is a new and effective approach to large-scale genome studies with high resolution, sufficient to detect deletions of 50 kb or duplications of 100 kb. CMA is now widely used in prenatal diagnosis and genetic testing. However, CMA cannot provide information on chromosome structure, whose alterations also may cause deletions or duplications. In general, for patients presenting multiple developmental abnormalities, genetic defects should be considered, and genetic testing (e.g., CMA and next generation sequencing) can be performed subsidiarily.

In summary, 4p16.2 microdeletion, which included part of EVC and EVC2 genes, may present mental and motor retardation with some mild features similar to EvC or WAD. This type of variation may be difficult to identify according to the clinical features; thus, genetic testing should be performed subsidiarily.

## Figures and Tables

**Table 1:** Clinical features of EvC, WAD, and the current patient.

Feature	EvC	WAD	The current patient
Postaxial polydactyly	+	+	—
Short stature	+	+	—
Short hands	+	—	+
Nail dysplasia	+	+	+, but mildly
Multiple frenula	+	+	—
Tooth abnormalities	+	+	—
Hypodontia	+	+	—
Motor delay	+, but rarely	—	+
Retarded brain development	+, but rarely	—	+
Mildly hypermyotonia	—	—	+
Hair change	+	—	+
Cardiac malformations	+	—	—
Abnormalities of other organs	+	—	—

EvC, Ellis-van Creveld syndrome; WAD, Weyers acrofacial dysostosis; +, present; —, not present.

## Data Availability

No data were used in this study.
